# Congenital adrenal hyperplasia with cholestatic jaundice: a case report

**DOI:** 10.1186/1687-9856-2015-S1-P55

**Published:** 2015-04-28

**Authors:** DoThi Thanh Mai, Vu Chi Dung, Bui Phuong Thao, Nguyen Ngoc Khanh, Can Thi Bich Ngoc, Nguyen Phu Dat

**Affiliations:** 1 National Hospital of Pediatrics, Hanoi, Vietnam

## 

Congenital adrenal hyperplasia (CAH) describes a group of autosomal recessive disorders, each of which involves a deficiency of an enzyme involved in the synthesis of cortisol, aldosterone, or both. Classic CAH is rare, about 1 case per 16,000 population. However CAH with cholestatic jaundice is extremely rare.

A 23 days old boy presented with vomiting, persistent jaundice. He was born at term, and his birth weight was 3 kg. In family history, no liver or endocrine disease was reported.

On examination, his weight was 3 kg; his height was 51 cm; jaundice, hyperpigmentation, dehydration, no hepatomegaly. Strength of his pennis was 3 cm; 2 testis were in the scrostum with volume of 1 ml.

Investigation showed : electrolyte imbalance Na^+^ 110 mmol/l, K^+^ 7.3 mmol/l, Cl^-^ 80mmol/l, 17 OHP 111 ng/ml, Testosteron 36.36 nmol/l, cholestatic jaundice : total bilirubin 114.6 mcmol/l, direct bilirubin 75.5 mcmol/l,GOT 40 UI/l, GPT 25UI/l, GGT 142.76 UI/l. The markers for viral hepatitis were negative. Abdominal ultrasound was normal.

He was diagnosed of CAH and treated with hydrocortisone and fludrocortisone. After 1 month of treatment, jaundice disappears and electrolyte is normalized

Written informed consent was obtained from the patient for publication of this Case report (and any accompanying images). A copy of the written consent is available for review by the Editor-in-Chief of this journal.

